# Distribution of *bla*_OXA-10_, *bla*_PER-1_, and *bla*_SHV_ genes in ESBL-producing *Pseudomonas aeruginosa* strains isolated from burn patients

**DOI:** 10.1038/s41598-023-45417-4

**Published:** 2023-10-26

**Authors:** Reem F. Polse, Haval M. Khalid, Wijdan M. S. Mero

**Affiliations:** 1 Department of Biology, Faculty of Science, University of Zakho, Kurdistan Region, Zakho, 42002 Iraq; 2 College of Science, Nawroz University, Kurdistan Region, Duhok, 42001 Iraq

**Keywords:** Microbiology, Molecular biology

## Abstract

*Pseudomonas aeruginosa* is resistant to a wide range of extended spectrum-lactamases (ESBLs) antibiotics because it produces several kinds of ESBLs. The goal of the current investigation was to identify the bacteria that produce extended spectrum -lactamases and the genes that encode three different ESBLs, such as *bla*_OXA-10_, *bla*_PER-1_ and *bla*_SHV_ genes in *Pseudomonas aeruginosa* isolated from burn patients. In this investigation, 71 *Pseudomonas aeruginosa* isolates were isolated from burn wounds in Burn and Plastic Surgery Hospital, Duhok City between July 2021 to June 2022. For the purpose of finding the *bla*_OXA-10_, *bla*_PER-1_, and *bla*_SHV_ ESBL expressing genes, Polymerase Chain Reaction (PCR) was used. Among 71 *Pseudomonas aeruginosa* isolates, 26.36% (29/71) were isolated from males and 38.18% (42/71) from females, and 76.06% (54/71) of the isolates were multidrug resistant. They exhibited higher resistance against Piperacillin with resistance rates of 98.59%. Among the ESBL-producing isolates tested, *bla*_OXA-10_ was found in 59.26% (32), *bla*_PER-1_ was found in 44.44% (24), and *bla*_SHV_ was found in 11.11% (6). All isolates must undergo antimicrobial susceptibility testing because only a few numbers of the available antibiotics are effective for the treatment of this bacterium. This will prevent the development of resistance in burn units and aids in the management of the treatment plan.

## Introduction

Burns can be induced by a variety of factors, including as heat, radiation, electricity, friction, or chemical contact. Burn-related fatalities are estimated to be 180,000 every year, due to the fact that low- and middle-income developing countries account for the majority of these deaths^1^. Burn wounds are an ideal environment for opportunistic organisms of both endogenous and foreign origin to flourish in infected sites. Infections can develop in burn patients for a variety of reasons, including exposed body surfaces, immunocompromised states, invasive operations performed in the hospital, and extended hospital stays. Invasive infection is influenced by both patient-related parameters like age, total body surface area (TBSA), and burn wound depth as well as microbiological organism-related ones like organisms’ variety and number, enzyme/toxin production, and motility^2^. For patients with severe burns covering more than 40% of their (TBSA), sepsis due to burn wound infection or other infection complications and/or inhalation injury accounts for 75% of all deaths^3^.

*Pseudomonas aeruginosa* is a non-fermentative, aerobic, Gram-negative rod that is widely present in the environment. It is one of the most significant opportunistic pathogens that has been linked to both community- and hospital-acquired illnesses, including nosocomial infections, otitis media, burns, and respiratory tract infections^4^. *Pseudomonas aeruginosa* infections are occurring more often, and isolates that are multidrug-resistant (MDR) are becoming more common in patients who are hospitalized^5,6^. This bacterium has the capacity to infect almost all tissues, leading to a significant increase in morbidity and mortality^7^. *Pseudomonas aeruginosa* is physiologically resistant to many antibiotics and disinfectants, and it is resistant to extended-spectrum antibiotics. Because of the synthesis of numerous kinds of extended spectrum-lactamases, cephalosporins such as ceftazidime, ceftriaxone, and cefotaxime (ESBLs). The prevalence of ESBLs in *P. aeruginosa* has risen^8–10^. The Ambler classification that found in ESBLs Gram-negative bacteria divide them into four groups: A, B, C, and D. These ESBLs play a significant role in the development of ß-lactam antibiotic resistance^11^. Mobile genetic elements (MGEs) such as plasmids, transposons, insertion sequences, integrons, and bacteriophages contribute to the dissemination of various ESBL-encoding genes. MGEs can move themselves and/or genes from one location to another within the cell or be transferred from cell to cell horizontally by conjugation, transformation or, in the case of bacteriophages, by transduction^12^.

*Pseudomonas aeruginosa* possesses a number of ESBLs, including those of the Ambler class A, which contain a variety of enzymes, such as Pseudomonas extended resistance *bla* (PER-1). In 1993, a Turkish patient being treated at a French hospital provided the first proof of PER-1, which only *P. aeruginosa* produces^13^. Displaying resistance to Cephalosporins and inhibition to Clavulanate, this enzyme hydrolyzed most Penicillins well and Cephalosporins including Cefalotin, Cefoperazone, Cefuroxime, Ceftriaxone, and Ceftazidime. PER-1 did not hydrolyse Oxacillin, Cephamycins or Imipenem. PER enzymes are most commonly found in isolates from Turkey and Mediterranean countries^14^.

*Pseudomonas aeruginosa* is the documented source of oxacillinase (OXA type), a class D -lactamase that hydrolyzes Oxacillin, but it has also been found in numerous other gram-negative bacteria, including Enterobacteriaceae. In general, OXA-type enzymes are a diverse category that exhibits variation in amino acid sequences and substrate profiles. However, it has been shown that a number of OXA-type variations hydrolyze cephalosporins, cephems, and/or monobactams. According to a recent review, there are 27 oxacillinase enzymes described as extended-spectrum. These enzymes’ substrates include third- and/or fourth-generation Cephalosporins in addition to Penicillins and early Cephalosporins. Most extended-spectrum oxacillinases derive from OXA-10 and OXA-2. The OXA-10 derivatives include OXA-11, OXA-13, OXA-14, OXA-16, OXA-17, OXA-19 and OXA-28^15^. In general, -lactamase inhibitors have no effect on OXA-type enzymes^16^. The occurrence of SHV-type ESBLs has been recorded in a number of European nations, including Austria, France, Italy, and Greece, as well as the United States and Australia. Perhaps more so than any other form of ESBL, they will be found in clinical isolates^17^.

The goal of the current investigation was to identify the patterns of antibiotic susceptibility against various antibiotics because *P. aeruginosa* is becoming increasingly resistant to multiple ESBLs. In addition, genes encoding *bla*_OXA-10_, *bla*_PER-1_, and *bla*_SHV_ were analyzed in clinical isolates of *P. aeruginosa* from infected hospitalized burn patients to assess their prevalence.

## Results

In this current study, a total of 110 burn patient samples of both genders and different ages were collected from July 2021 to May 2022. From 110 samples, *P. aeruginosa* was the predominant pathogenic bacteria, and 71 (64.55%) of them were confirmed phenotypically and molecularly by the species-specific gene (16S rDNA). Phenotypically, *P. aeruginosa* was identified on MacConkey, Cetrimide, Nutrient, and Blood agars as well as Gram stain and biochemical tests (Oxidase, Citrate, Catalase, TSI). In addition, all isolated *P. aeruginosa* were genotypically confirmed by the species-specific gene (16S rDNA).

Among 71 *P. aeruginosa*, 26.36% (29/71) were isolated from males and 38.18% (42/71) from females. There was a statistically non-significant (P > 0.05) difference between both genders. Most isolates were collected from ages between 21 and 30 years (13.64%). Statistical analysis revealed that there were significant (P < 0.04) differences across age groups. The demographic information for 71 positive patients from whom *P. aeruginosa* was isolated are displayed in Table 1. The most frequent types of burn wounds were those caused by flame, accounting for 38.18% (42/110) of the patients; electric burns affected 2.73% (3/110) of the patients. Statistical analysis showed highly significant (P < 0.01) differences in the causes of burn. Patients with second-degree burns had a higher risk of contracting infection (45.45%) than third- and mix-degree burns (6.36% and 12.73%) respectively. Statistical analysis revealed the presence of highly significant (P < 0.01) differences between degrees of burns. Patients with TBSA burns between 20 and 40 percent (48.18%) had a higher risk of infection than those with burns between 20 and 40 percent (7.27% and 9.09%), but these differences were statistically non-significant (P > 0.05) (Table 2).

**Table 1:** The relationships between burn causes among both genders and different ages.

Demographic characters	Factors	No. of samples	No. of positive isolates with (%)	Odd (ratio 95% CI)	P value
Gender	Female	62	42 (38.18%)	1.376 (0.6099–3.114)	0.5469
	Male	48	29 (26.36%)		
	Total	110	71 (64.55%)		
Age group	1 month–10 Y	29	13 (11.82%)	Reference group	–
	11–20	22	12 (10.90%)	0.6771 (0.2077–2.076)	0.5771
	21–30	23	15 (13.64%)	0.4333 (0.1510–1.314)	0.1707
	31–40	18	14 (12.73%)	0.2321 (0.07210–0.9406)	0.0359
	41–50	10	9 (8.18%)	0.09028 (0.007819–0.6224)	0.0240
	51–60	4	4 (3.64%)	0.000 (0.000–1.069)	0.1026
	61–70	3	3 (2.73%)	0.000 (0.000–1.083)	0.2258
	71–80	0	0	–	
	81–90	1	1 (0.91%)	0.000 (0.000–7.875)	0.4667
	Total	110	71 (64.55%)	–	
Cause of burn	Flame	76	42 (38.18%)	Reference group	–
	Scald	22	17 (15.45%)	0.3633 (0.1370–1.058)	0.0841
	Hot liquids	9	9 (8.18%)	0.000 (0.000–0.5348)	0.0098
	Electric	3	3 (2.73%)	0.000 (0.000–1.501)	0.2551
	Total	110	71 (64.55%)	–	

*Cause of burn: Chi-square = 11.01, 3 and *P value 0.0117.

*Age group: Chi-square = 14.50, 7 and *P value 0.0430.

**Table 2:** Distribution of burnt TBSA and depth (degree) in relation to infection.

Variables and their patterns distribution		Total no	Infected No. with (%)	Chi-square	P value
Degree of burn	Second degree	65	50 (45.45%)	17.73	0.0001
	Third degree	8	7 (6.36%)		
	Mixed	37	14 (12.73%)		
	Total	110	71 (90.91%)		
TBSA group	< 20	17	8 (7.27%)	3.410	0.1818
	20–40	76	53 (48.18%)		
	> 40	17	10 (9.09%)		
	Total	110	71 (90.91%)		

Antimicrobial susceptibility test revealed that 76.06% (54/71) *P. aeruginosa* isolates were multidrug-resistant (MDR), showing resistance to β-lactams, aminoglycosides, and/or fluoroquinolones (at least three different types of antimicrobial medicines).

All of the isolated *P. aeruginosa* in the present study were sensitive to Colistin (100%). While, they displayed strong levels of resistance against Meropenem, Ceftazidime, Cefepime, and Piperacillin at rates of 74.65%, 84.50%, 85.92%, and 98.59% respectively. However, they experienced moderate resistance to Imipenem, Amikacin, Ciprofloxacin, Levofloxacin, and Gentamicin at rates of 60.56%, 63.38%, 64.79%, 67.61%, and 69.01%, respectively (Table 3).

**Table 3:** Susceptibility patterns to antimicrobial agents.

Antimicrobial agents	R (%)	I (%)	S (%)
CL	0	0	71 (100)
IMP	43 (60.56)	1 (1.41)	27 (38.03)
CIP	46 (64.79)	3 (4.23)	22 (30.99)
LEV	48 (67.61)	1 (1.41)	22 (30.99)
CN	49 (69.01)	2 (2.82)	20 (28.17)
AK	45 (63.38)	8 (11.27)	18 (25.35)
MEM	53 (74.65)	3 (4.23)	15 (21.13)
CAZ	60 (84.50)	1 (1.41)	10 (14.08)
FEP	61 (85.92)	2 (2.82)	8 (11.27)
PI	70 (98.59)	0	1 (1.41)

R, resistance; S, sensitive; I, intermediate; CL, colistin; IMP, imipenem; CIP, ciprofeloxacin; LEV, levofloxacin; CN, gentamicin; AK, amikacin; MEM, meropenem CAZ, ceftazidime; FEP, cefepime; PI, piperacillin.

Using PCR amplification, the prevalence of the antibiotic resistance genes *bla*_OXA-10_, *bla*_PER-1_, and *bla*_SHV_ in the ESBL-producing *P. aeruginosa* isolates were examined and confirmed by agarose gels. Among the ESBL genes, *bla*_OXA-10_, and *bla*_PER-1_ were more abundant at 59.26% (32), and 44.44% (24), respectively, and the *bla*_SHV_ had the lowest abundance of 11.11% (6). The main point of interest is that 14 of the isolated *P. aeruginosa* strains had both *bla*_OXA-10_, and *bla*_PER-1_ genes, and 6 strains had *bla*_OXA-10_, and *bla*_SHV_. Also, *bla*_OXA-10,_ *bla*_PER-1,_ and *bla*_SHV_ genes had 5 strains. Furthermore, four strains possess *bla*_PER-1,_ and *bla*_SHV._ (Table 4).

**Table 4:** Representation of the antibiotic resistance of ESBL-producing *P. aeruginosa.*

ESBL-producing *P. aeruginosa* genes	No. of strains
*bla*_OXA-10_	32
*bla*_PER-1_	24
*bla*_SHV_	6
*bla*_OXA-10,_ *bla*_PER-1,_ and *bla*_SHV_	5
*bla*_OXA-10,_ and *bla*_PER-1_	14
*bla*_OXA-10_ and *bla*_SHV_	6
*bla*_PER-1_ and *bla*_SHV_	4

The isolates carrying the *bla*_OXA-10_ gene were completely resistant to Piperacillin, Cefepime, and Ceftazidime, and showed high to moderate resistance to other tested antibiotics, while isolates with *bla*_PER-1_ gene, were 100% resistant to Piperacillin, Ceftazidime, and Meropenem, and showed high to moderate resistance to other tested antibiotics, finally isolates with *bla*_SHV_ gene were 100% almost resistant to all antibiotics. We can show all isolates with *bla*_OXA-10,_ *bla*_PER-1,_ and *bla*_SHV_ genes were 100% sensitive to Colistin (Table 5).

**Table 5:** Frequency of ESBLs producing strains and susceptibility patterns to antimicrobial agents in relation to *bla*_OXA-10_, *bla*_PER-1_ and *bla*_SHV**.**_

Antimicrobial agents	*bla*_OXA-10_ positive (32)			*bla*_PER-1_ positive (24)			*bla*_SHV_ positive (6)		
	R (%)	I (%)	S (%)	R (%)	I (%)	S (%)	R (%)	I (%)	S (%)
CL	_0_	_0_	_32 (100)_	_0_	_0_	_24 (100)_	_0_	_0_	_6 (100)_
IMP	_27 (84.38)_	_1 (3.12)_	_4 (12.5)_	_21 (87.5)_	_0_	_3 (12.5)_	_5 (83.33)_	_0_	_1 (16.67)_
CIP	_29 (90.62)_	_2 (6.25)_	_1 (3.12)_	_21 (87.5)_	_1 (4.17)_	_2 (8.33)_	_6 (100)_	_0_	_0_
LEV	_30 (93.75)_	_1 (3.12)_	_1 (3.12)_	_22 (91.69)_	_0_	_2 (8.33)_	_6 (100)_	_0_	_0_
CN	_27 (84.38)_	_1 (3.12)_	_4 (12.5)_	_19 (97.17)_	_1 (4.17)_	_4 (16.67)_	_4 (66.67)_	_0_	_2 (33.33)_
AK	_25 (78.13)_	_5 (15.63)_	_2 (6.25)_	_20 (83.33)_	_3 (12.5)_	_1 (4.17)_	_5 (83.33)_	_1 (16.67)_	_0_
MEM	_31 (96.88)_	_0_	_1 (3.12)_	_24 (100)_	_0_	_0_	_5 (83.33)_	_0_	_1 (16.67)_
CAZ	_32 (100)_	_0_	_0_	_24 (100)_	_0_	_0_	_6 (100)_	_0_	_0_
FEP	_32 (100)_	_0_	_0_	_23 (95.83)_	_1 (4.17)_	_0_	_6 (100)_	_0_	_0_
PI	_32 (100)_	_0_	_0_	_24 (100)_	_0_	_0_	_6 (100)_	_0_	_0_

R, resistance; S, sensitive; I, intermediate; CL, colistin; IMP, imipenem; CIP, ciprofeloxacin; LEV, levofloxacin; CN, gentamicin; AK, amikacin; MEM, meropenem CAZ, ceftazidime; FEP, cefepime; PI, piperacillin.

## Discussion

Recently, ESBL-producing *P. aeruginosa* has become a significant source of infections in healthcare settings, particularly in immunocompromised individuals and burn victims^18^. Because the surface of moist wounds provides a habitat that is suited for their existence, it thrives in the clinical setting, proving that this environment is perfect for their colonization. Because of the high levels of resistance to the most often given antibiotics in hospital settings, the treatment of infections brought on by these multidrug-resistant organisms is becoming more difficult^19^.

Our study revealed the prevalence of *P. aeruginosa* isolates among burn patients at the Duhok Burn Hospital, at a rate of 64.55% (71/110), a similar finding was made in an Algerian study, where the rate was recorded at 62%^20^. On the other hand, another study conducted in Iraq recorded a high prevalence of *P. aeruginosa* isolates at 97.6%^21^. however, results from other studies from other nations showed lower prevalence rates, including Morocco (15.1%)^22^, and Egypt (19.8%)^23^. This difference might be attributed to antibiotics abuse, different hospital strategies for the management of infections, hygiene, and geographic climatic.

The current study showed the highest rate of 38.18% from flame burn followed by scald burn (15.45%). Regarding gender and age, females showed the highest rate of burn wounds than males (38.18% vs 26.36%), with the ages of 21–30 showing the highest rate (13.64%). These results are somewhat similar to the study conducted in Basra/Iraq^24^ they showed that females had a higher proportion of burns than males (57.5% vs. 19.16%), with flame burns accounting for 76.6%, followed by scald burns (19.1%). Additionally, studies from Iraq and Iran have reported somewhat lower rates; 57% in the Iraqi city of Suleimani^25^, 54.84%, and 56% in Iran^1,26^ respectively. Due to the fact that most flame injuries occur at home and that most women in our society perform daily household tasks like heating and cooking in areas of the kitchen where there is a greater danger of flame burns, females were significantly more likely than males to sustain flame burns.

While a study conducted in Suleimani/Iraq showed the highest infection in males (56.2%) with scald (Hot water) being the highest (72.5%) group involved, followed by flame (22.8%)^27^. According to this research's findings, the highest rate 45.45% were second-degree burn, followed by 12.73% of mixed burn infections and only 6.36% had third-degree burns. Similarly, Al-Aali et al.^3^ revealed that 72.1% of burn patients had second-degree burns. According to the present study observations, patients who had TBSA burns were 20–40% more likely to contract an infection (48.18%), this finding is consistent with the research conducted by Rashid et al.^27^ in Suleimani/Iraq found infection rates were higher in patients with TBSA burns of 20–40% compared to those with burns of 40%. The higher the total body surface area damaged by the thermal assault the higher the potential for the bacteria to colonize and proliferate increasing the wound thickness and depth making way to the bloodstream involvement^28^.

Regardless to the etiology of the burns, female patients over the age of ten years were the most prevalent patient demographics linked to a higher prevalence of *P. aeruginosa* infection. In the present investigation, 76.06% of the patients had MDR *P. aeruginosa* infection which is higher than that reported by other studies in Iran (16.5–41%), Iraq (12.4%)^29–31^ , Brazil (71.4%) and Egypt (70%)^32,33^. On the other hand, another study in Iran found a much higher rate (89.24%) of patients infected with MDR *P. aeruginosa*^26^. Worldwide increasing of MDR *P. aeruginosa* could be attributed to improper use of antibiotics in hospitals and communities in addition to the accumulation of a variety of resistance mechanisms^34^.

In the current investigation, 100% of the isolates demonstrated sensitivity to Colistin. In contrast, a substantially lower percentage of Colistin-sensitive individuals (53.1%) were reported by Jalil et al.^24^ in their investigation in Iraq. Another study in Iraq also reported resistance to colistin at a rate of 7.4%^21^. However, susceptibility to Colistin remains vastly high against *P. aeruginosa* approaching 100% in most countries in the area of the Middle East and North Africa^35^.

In this study, 60.56% of *P. aeruginosa* isolates were resistant to Imipenem, which is higher than that reported by a study conducted in Iran at a rate of 41.3%^17^ and Iraq at a rate of 47%^36^, and lower than that reported by studies conducted in Iran at a rate (83.9%)^26^, in Iraq 68.40%^21^. Multifactorial agents, such as increased carbapenemase production, oprD mutation, AmpC, and efflux pumps overexpression, a shift in drug target sites, and other mechanisms, are contributing to *P. aeruginosa* increasing resistance to carbapenems^37^.

The current study reported the highest resistance of *P. aeruginosa* to Piperacillin (98.59%). This finding is higher than previous studies conducted in Iran 74.8%^38^ and in South Africa, 94%^39^. These findings raise a major concern that requires Health Authorities to urgently work on finding rapid and accurate diagnostic procedures and regulating the dispensing of antibiotics, in addition to tightening microbiological control systems in hospitals.

ESBLs are one of the most common sources of ß-lactam antibiotic resistance in Gram-negative bacteria. These enzymes are plasmid-encoded ß-lactamases that have been discovered in *Klebsiella pneumoniae* and *Escherichia coli*, as well as clinical isolates of Enterobacteriaceae and *Pseudomonas*^19^. In the current study, the genes linked to *bla*_OXA-10_, *bla*_PER-1_, and *bla*_SHV_ were found in 59.26% (32), 44.44% (24), and 11.11% (6) of the ESBL-producing *P. aeruginosa* isolates. The level of resistance was even higher in isolates carrying the OXA-10 gene. The results were consistent with a study conducted in Iran by Farshadzadeh^40^, which used the same primers for the OXA-10 and PER-1 genes and produced a band of the same molecular weight. Another study conducted in Shiraz, Iran reported a rate of 76.2% *bla*_OXA-10,_ and 40% of resistant isolates contain *bla*_PER-1_-related genes^13^. The prevalence of the *bla*_SHV_ gene in the current study is consistent with the rate of 10.7% reported by Peymani et al.^17^ in Iran.

Different environments may have a different distribution of ESBL-resistant bacteria, as evidenced by the varying prevalence of ESBL-encoding genes. In addition, the prevalence of *bla*_PER-1_ in Ahvaz, Iran, was 62.75%, which was higher than the prevalence of *bla*_PER-1_ in Italy (34.61%)^41^, Hungary (1.3%), and Belgium (2%), respectively^42^, but less than the incidence of *bla*_PER-1_ in Turkey (86.75%)^43^. The results of our study showed a high prevalence of the ESBL-producing *P. aeruginosa* isolates in burn patients, which is an alarming sign and should be taken into consideration because increasing of the antimicrobial-resistant bacteria isolated from burn patients is an important issue.

## Methods

### Sample collection

In the current study, one hundred ten clinical samples were collected from patients attending Burn and Plastic Surgery Hospital in Duhok City, Iraq over a period from July 2021 to May 2022. These samples were collected from hospitalized patients of both genders and different ages (1 month–90 years). The clinical samples were collected from infected burn wounds at the same time the dressings were changed using cotton disposable swabs and transported into the sterile medium in plastic bottles.

### Isolation and Identification of *Pseudomonas aeruginosa*

After taking the samples with sterile cotton swabs and placed in a tube containing 5 ml of brain heart infusion broth, the samples were incubated at 37 °C for 24 h. Then the samples were cultured by a sterile loop using the streak method on, MacConkey agar. After the incubation for 24 h at 37 °C, the subculture of the pure colonies of suspected *P. aeruginosa* were done every 48 h on Blood agar, Cetrimide agar, and Nutrient agar plates. Initial diagnosis of isolates was made on the basis of Gram’s staining of culture, hemolysis on blood agar, pigment production, and the smell in cultures. Based on the morphological and biochemical characteristics of these pure colonies were identified according to a study by^44^.

### Antimicrobial susceptibility test

All isolated *P. aeruginosa* were subjected to antibiotic susceptibility test using Kirby–Bauer disk diffusion method by spreading the inoculated sterile swab on Muller–Hinton agar and incubated overnight aerobically at 37 °C according to^45^. Ten antibiotics were used, supplied by (Bioanalyses, Turkey). The tested antimicrobial agents included: Piperacillin (PI; 100 mg), Ceftazidime (CAZ; 30 mg), Cefepime (CPM; 30 mg), Imipenem (IPM; 10 mg), Meropenem (MEM; 10 mg), Amikacin (AK; 30 mg), Gentamicin (CN; 10 mg), Levofloxacin (LEV; 5 mg), Ciprofloxacin (CIP; 5 mg), Colistin (CL; 10 mg). The diameter of the inhibition zone around antibiotic disks was measured according to the Clinical and Laboratory Standards Institute^46^.

### Bacterial DNA extraction from *P. aeruginosa*

Genomic DNA was extracted from bacterial isolates using High yield DNA Purification Kit according to the manufacturer’s instructions (Genomic DNA mini kit, Favorgen, Taiwan). Bacterial DNA quality was achieved by (NanoDrop™ One UV–Vis Spectrophotometer, Thermo Fisher Scientific, Waltham, MA, USA) and then stored in a freezer at – 20 °C, ready to be used for PCR. Different primers were used for amplification of these genes as described in Table 6. Purified DNA was used for PCR amplification of 4 genes (Table 7). A species-specific primer for *P. aeruginosa*, 16S rDNA gene. After confirming *P*. *aeruginosa*, 3 primers were used to detect ESBL genotypes, *bla*_OXA-10_, *bla*_PER-1_, and *bla*_SHV_ β-lactamase genes.

**Table 6:** Molecular weights of the genes and sequences of the primer used.

Primer name	Primer sequence (5′–3′)	Detected gene	Molecular weight	References
16S rDNA	F: GGGGGATCTTCGGACCTCA R:TCCTTAGAGTGCCCACCCG	16S rDNA	956	^47^
OXA-10	ABD1 _F: TAT CGC GTG TCT TTC GAG TA ABD4 _R: TTA GCC ACC AAT GAT GCC	*bla*_OXA-10_	760 bp	^48^
PER-1	F: ATG AAT GTC ATT ATA AAA GC R: TTA ATT TGG GCT TAG GG	*bla*_PER-1_	927 bp	^49^
SHV	F:TCGTTATGCGTTATATTCGCC R:GGTTAGCGTTGCCAGTGCT	*bla*_SHV_	861 bp	^50^

**Table 7:** PCR program that applies in the thermo-cycles.

Gene name	Temperature (°C)/time				
	Initial denaturation	Cycling condition			Final extension
		Denaturation	Annealing	Extension	
16S* rDNA*	95 °C; 2 min; 1 cycle	94 °C; 20 s	54 °C; 20 s	72 °C; 40 s	72 °C; 5 min 1 cycle
		25 cycle			
*bla*_OXA-10_	95 °C; 5 min 1 cycle	94 °C; 1 min	57 °C; 1 min	72 °C; 1 min	72 °C; 5 min 1 cycle
		30 cycle			
*bla*_PER-1_	95 ℃; 5 min 1 cycle	94 °C; 1 min	48 °C; 1 min	72 °C; 1 min	72 °C; 5 min 1 cycle
		30 cycle			
*bla*_SHV_	94 °C; 5 min 1 cycle	94 °C; 30 s	58 °C; 30 s	72 °C; 1 min	72 °C; 10 min 1 cycle
		30 cycle			

The amplification of DNA for each gene was carried out in PCR tubes containing 10 µl of PCR master mix (ADDBIO.INC, South Korea), 3 µL of DNA, 5 µL of distilled deionized water, and the forward and reverse primers, totaling 1 µL of each primer (10 pmol/µL), to make a final volume of 20 µL. The amplification conditions of each gene are described in Table 7.

The DNA lengths of each fragment produced with these 4 primers were confirmed by running 5 µL of each PCR product on 1.5% agarose gels in 1 × TAE buffer and added 8 µL of Safe Gel stain Dye (Guangzhou Dongsheng Biotech Co., Ltd., Guangzhou, China) and the electrophoresis was performed at 85 V for 45 min. The agarose gel was visualized under UV radiation (Cleaver Scientific Ltd., Rugby, UK). The images of DNA bands were captured, and the estimated amplicon size were compared with the 1500–100 bp DNA ladder (Guangzhou Dongsheng Biotech Co., Ltd., Guangzhou, China)^51^.

### Ethics declarations

The study protocol was received and approved by the Duhok Directorate General of Health, Directorate of Planning, Scientific Research Division, Institutional Ethics Committee (approval No. 13072021-7-10).

### Approval for human experiments

1. The study was approved by the Duhok Directorate General of Health, Directorate of Planning, Scientific Research Division, Institutional Ethics Committee (approval No. 13072021-7-10).
2. I confirm that all experiments were performed in accordance with relevant named guidelines and regulations.

### Informed consent statement

The request for verbal consent is part of the original approval (with reference number 13072021-7-10 on 21 May 2023) and the researcher was not asked for written informed consent.

### Statistical analysis

Statistical analysis was performed using GraphPad Prism version 9.3.1 (471). The chi-square test and Odd (ratio 95% CI) test were performed to determine statistical significance at *P-*value of less than 0.05.

## Conclusion

In conclusion, the current study's high proportion of ESBL-producing *P. aeruginosa* highlights a serious health issue that requires further attention. All isolates must undergo an antimicrobial susceptibility test prior to therapy because there are only a small number of medications that are effective against this bacterium. The execution of this test reduces the indiscriminate use of antibiotics and resistance development in burn units and aids in the management of treatment plan.

## Data Availability

All data supporting the present work are available from the corresponding author upon reasonable request.
